# Construction and demolition waste as recycled aggregate for environmentally friendly concrete paving

**DOI:** 10.1007/s11356-021-15849-4

**Published:** 2021-09-10

**Authors:** Manuel Contreras Llanes, Maximina Romero Pérez, Manuel Jesús Gázquez González, Juan Pedro Bolívar Raya

**Affiliations:** 1 Department of Sociology, Social Work and Public Health, Research Centre for Natural Resources, Health and Environment (RENSMA), University of Huelva, 21007 Huelva, Spain; 2 Department of Integrated Sciences, Research Centre for Natural Resources, Health and Environment (RENSMA), University of Huelva, 21007 Huelva, Spain; 3 Department of Materials, Instituto de Ciencias de la Construcción Eduardo Torroja (IETcc-CSIC), 28033 Madrid, Spain; 4 Department of Applied Physics, Marine Research Institute (INMAR), University of Cádiz, 11510 Cádiz, Spain

**Keywords:** Civil engineering, Construction and demolition waste, Recycled aggregate, Environmentally friendly concrete, Green paving units

## Abstract

Recycled aggregates (RA) from construction and demolition waste (CDW) instead of natural aggregates (NA) were analysed in the manufacture of new eco-friendly concrete. Fine (FRA) and coarse (CRA) recycled aggregates were used in different percentages as substitutes of natural sand and gravel, respectively. The results revealed that the use of RA in percentages of up to 50 wt.% is feasible. Additionally, RA were used to produce paving blocks in accordance with industrial requirements. Thus, values of water absorption lesser than 6.0% and tensile strength upper than 3.6 MPa were obtained, which are similar to those of a reference sample and within the limit values established by the regulations. These results were achieved by reducing the incorporation of cement, thereby saving production costs and minimizing environmental impact.

## Introduction

The increase in population in emerging countries, together with the increase in residential development and the need for infrastructure improvements in developed countries, has meant that the construction sector has grown at a moderate rate in recent years and that the long-term outlook at the global level is positive. Thus, in the next decade, the construction sector is expected to grow above the growth of the world’s gross domestic product (GDP) according to the Eurostat (2021). Within this sector, the demolition and rehabilitation works occupy an important parcel since the shortage of space in large cities often leads to a choice of demolition or rehabilitation works before undertaking a new construction project. However, this activity generates large amounts of construction and demolition waste (CDW). In 2016, 3.5×10^8^ tons CDW were generated in Europe (Eurostat 2021), being the largest waste stream and representing almost a third of the total waste originated (EU Construction and Demolition Waste Management Protocol 2016). In turn, the generation of CDW in the USA in 2015 was 5.8 ×10^8^ tons according to the CDRA (2021), which is considered to constitute between 20 and 30% of the overall amount of municipal solid waste generated (Martín-Morales et al. 2011).

CDW are comprised of a broad range of materials, such as concrete, ceramics, brick, rock, metal, plaster, wood, glass, soil and asphalt (Medina et al. 2015; Villoria et al. 2011). The components of CDW are mostly non-hazardous. However, they may also contain harmful materials to both human health and the environment (U.S. EPA 530-R-98-010 1998). In this regard, some studies highlight that certain CDW have significant amounts of leachable heavy metals, specifically arsenic and lead (Tolaymat et al. 2004). Moreover, the European Waste Catalogue (EWC) and Hazardous Waste List classify this waste as inert with code 17 01 07 (Commission Decision 2014/955/EU of 18 December 2014; Directive 2000/532/EC of 3 May 2020).

Currently, most developed countries have no specific recovery or recycling plan for CDW, so their management is limited to of controlled landfill disposal. The situation in developed countries is even worse, as most CDW often end on illegal landfills or dumped in urban areas or on roads, with the consequent environmental problem. In view of this reality, in recent years, governments have implemented environmental policies to establish a regulatory framework that allows new recycling strategies. In order to carry out a correct reuse and recycling of these CDW, the separation of the component such as wood, glass, gypsum and other undesired material is a crucial step. The different components of the CDW can be separated in situ at the construction or demolition site. However, the reality is the transport of the CDW to a recycling plant where the recycling, separation and recovery of its parts takes place (EU Construction and Demolition Waste Management Protocol 2016). In Europe, the average recycling rate of CDW is about 40%, but in Spain, this rate is much lower, and only 15% of the CDW generated is recycled (Villoria et al. 2011), which represents a significant gap from the 70% target established by the Integrated Waste Management Plan (Directive 2008/98/EC of 19 November 2008).

Nowadays, CDW has been successfully recycled as base and sub-base of road construction (Poon and Chan 2006a; Tavakoli Mehrjardi et al. 2020; Zhang et al. 2021), paving projects (Arulrajah et al. 2014; Gedik 2020; Özalp et al. 2016), footpaths projects (Arulrajah et al. 2013; Arulrajah et al. 2016; Pourkhorshidi et al. 2020) and pipe-bedding projects (Arulrajah et al. 2017; Rahman et al. 2014; Taha et al. 2002). Nevertheless, it is still necessary to develop new applications for the manufacture of new products, by developing originals processes, by seeking and finding new markets in order to absorb and reduce the vast volume of CDW worldwide production, and furthermore, to comply with the objective established by EU (Commission Decision 2014/955/EU of 18 December 2014; Directives 2000/532/EC of 3 May 2020, 2008/98/EC of 19 November 2008).

Consequently, previous studies using RA obtained from CDW have developed new applications mainly focused on the use of these sorts of materials as NA replacement in concrete production (Falek et al. 2017; Favaretto et al. 2017; Ibrahim et al. 2020; Idagu 2017; Lau et al. 2014; Martín-Morales et al. 2011; Rao et al 2007; Robalo et al. 2021; Tabsh and Abdelfatah 2009; Sharmal and Singla 2014; Silva et al. 2014; Yang et al. 2011) and concrete materials manufacturing, i.e., concrete bricks (Contreras et al. 2016; Devi et al. 2020; Poon et al. 2002; Rodríguez et al. 2016; Sadek et al. 2013; Silva et al. 2019) and concrete block (Kumar et al. 2020; Leiva et al. 2013; López et al. 2018; Poon et al. 2002, 2009; Sabai et al. 2013; Soutsos et al. 2011a). Moreover, several studies have focused on the application of the RA in the production of concrete paving but without promising results (Jankovic et al. 2012; Juan-Valdés et al. 2019; Poon and Chan 2006b, 2007; Soutsos et al. 2011b, 2012). Otherwise, high levels of RA recycling were achieved by some authors (Juan-Valdés et al. 2018; Kim 2021; López Gayarre et al. 2013; Wang et al. 2021), which occurs at the expense of significantly higher production costs, because of the addition of cement was increased, and cement is by far the most expensive material in concrete manufacturing. Therefore, it is necessary to minimise the consumption of cement, to achieve an economically realistic application.

This paper is part of an ambitious project that has been divided into two parts. The first part consisted in the substitution of NA by RA, which were obtained from a specific pre-treatment of CDW, in the concrete manufacturing. The second part, both the pre-treatment developed and the potential results obtained were validated on an industrial scale

In light of the above, this aim of this paper is to develop a process that allows simultaneously maximising the incorporation of RA and reducing the cement requirement necessary for the manufacture of concrete paving blocks, complying with the established requirements. Therefore, the physical and technological properties were determined and compared with standards materials according to the current regulations.

## Materials and methods

### Materials preparation

Representative sample of CDW was provided from a recycling plant in the region of Murcia (Spain). CDW was treated to obtain RA with physical and mechanical properties similar to those of NA. Hence, the RA were obtained through mechanical pre-treatment (crushing, grounding, sieving and removal of impurities) of the CDW. Firstly, the samples were crushed and passed through a sieve 12 mm. Secondly, the coarse fraction was ground and sieved through a 4.8-mm mesh sieve. Finally, two different grain size fractions were obtained, fine recycled aggregate (FRA) with a particle size < 4.8 mm, and coarse recycled aggregate (CRA) > 4.8 mm, which were used in this research. Regardless of the pre-treatment performed, RA showed a water absorption index higher than NA (sand and gravel) due to the presence of porous materials such as mortar, ceramic and clay (Poon and Chan 2006a, b; Yang et al. 2011). According to previous studies using RA obtained from CDW (Contreras et al. 2016; Ferreira et al. 2011), the pre-saturation (partial saturation of the superficial pores) of RA is an adequate method because of provide the RA with an extra amount of water to solve the problem of their higher porosity and thus to achieve concrete mixtures with the calculated water/cement ratio. Moreover, García-González et al. (2014) showed that this technique reduces water absorption during the cementation process, keeping the process water-free until the cement hydration and achieving an appropriate consistency and workability. It was found that the soaking of the RA in water for short intervals (about 3 min), in which the RA reached up to 50% of complete saturation (Contreras et al. 2016). This soaking time guaranteed a partial saturation (around 50%) obtaining plastic or soft slump consistencies with a minimum loss in the compressive strength of the final concrete (Contreras et al. 2016; García-González et al. 2014). Consequently, the pre-saturation of the aggregates could be adopted in ready mix concrete at the expense of some minor changes in the industrial manufacture process.

On the other hand, other materials necessary for concrete manufacturing (sand, gravel, cement and additive) were provided by the precast concrete company “Montalbán y Rodríguez S.L.”, placed in the region of Murcia (Spain). Eco-friendly mortars and paving blocks were prepared using a high-activity water reducing/superplasticising additive based on polycarboxylates MasterCast 731 supplied by BASF Company to improve consistency.

**Fig. 1:**
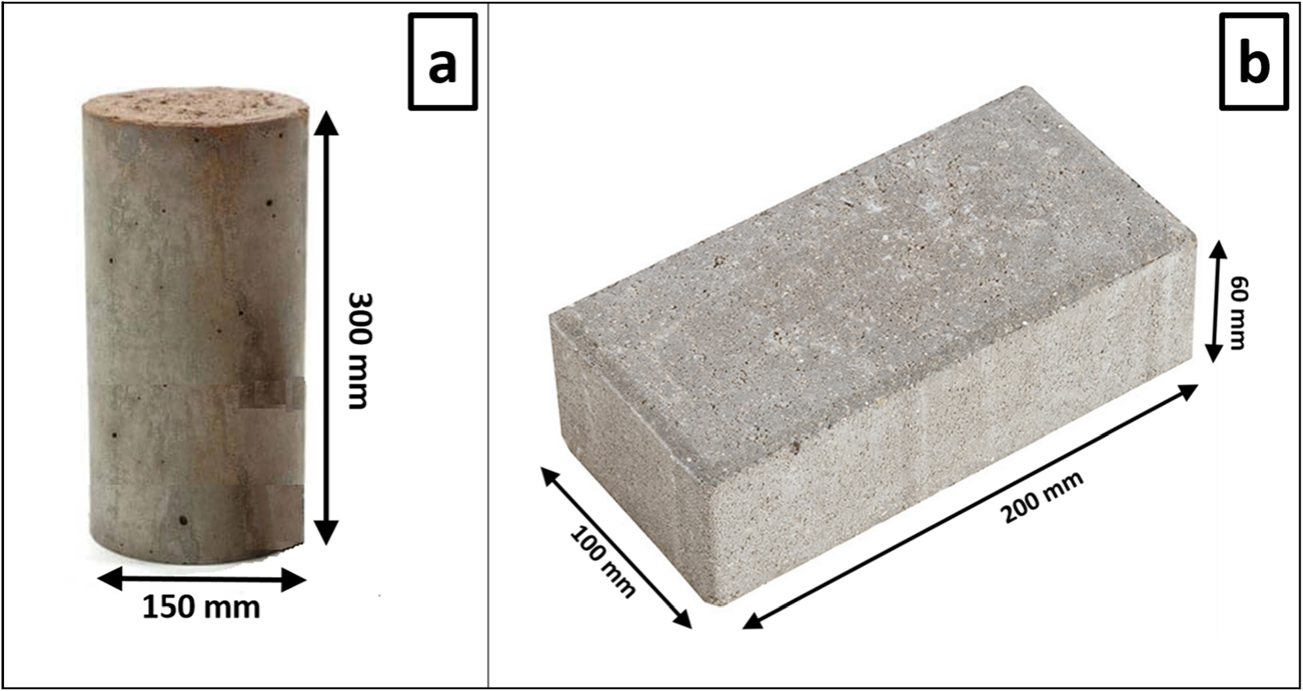
General appearance and dimensions of the cylindrical test specimens (**a**) and the paving blocks (**b**)

Different percentage compositions (Table 1) of RA were mixed with NA (sand and gravel), ordinary Portland cement (OPC) and additive. This OPC type I is characterised by a compressive strength of 32.5 N mm^−2^ and is composed of a mixture of clinker (97 wt.%) and natural gypsum (3 wt.%). The mixtures were moistened by spraying until a w/c ratio = 0.45 was reached, which is the optimum index for obtaining adequate consistency and a good workability used in the industrial process and which has been provided by the precast company. The moistened materials were homogenised and placed in steel moulds to obtain concrete cylindrical test specimens (*ϕ* = 150 mm, *h* ≈ 300 mm), which were finally vibrated as described in the UNE-EN 12390-3 standard (2020). Furthermore, hydrated mixtures were used in the manufacture of concrete pavements, which were pressed in duplicate, utilising a uniaxial hydraulic press at 30 tons in steel moulds to obtain cylindrical test specimens of 200 × 100 × 60 mm, in accordance with industrial requirements (Fig. 1a). The samples were stored in open air with a temperature of 22–30 °C and a relative humidity of 65–75% (UNE-EN 12390-3 2020).

**Table 1:** Code and composition of the different mixes tested (% by weight). Each sample was labelled as X-Y-Z where X is the percentage of FRA used as substituted of sand; Y is the percentage of CRA used as substituted of gravel; and Z is the percentage of cement added. The percentage of substitution of each fraction of RA (FRA and CRA) is shown in parentheses

Code	Sand	FRA (wt.%)	Gravel	CRA (wt.%)	Cement^*^	Water^**^
0-0-7	53.9	0 (0)	35.9	0 (0)	7.0	3.2
10-0-7	48.5	5.4 (10)	35.9	0 (0)	7.0	3.2
25-0-7	40.4	13.5 (25)	35.9	0 (0)	7.0	3.2
50-0-7	26.9	27.0 (50)	35.9	0 (0)	7.0	3.2
75-0-7	13.4	40.5 (75)	35.9	0 (0)	7.0	3.2
90-0-7	5.3	48.6 (90)	35.9	0 (0)	7.0	3.2
100-0-7	0	54.0 (100)	35.9	0 (0)	7.0	3.2
0-10-7	53.9	0 (0)	32.3	3.6 (10)	7.0	3.2
0-25-7	53.9	0 (0)	26.9	9.0 (25)	7.0	3.2
0-50-7	53.9	0 (0)	17.9	18.0 (50)	7.0	3.2
0-75-7	53.9	0 (0)	8.9	27.0 (75)	7.0	3.2
0-90-7	53.9	0 (0)	3.5	32.4 (90)	7.0	3.2
0-100-7	53.9	0 (0)	35.9	36.0 (100)	7.0	3.2
10-10-7	48.5	5.4 (10)	32.3	3.6 (10)	7.0	3.2
25-25-7	40.4	13.5 (25)	26.9	9.0 (25)	7.0	3.2
50-50-7	26.9	27.0 (50)	17.9	18.0 (50)	7.0	3.2
75-75-7	13.4	40.5 (75)	8.9	27.0 (75)	7.0	3.2
90-90-7	5.3	48.6 (90)	3.5	32.4 (90)	7.0	3.2
100-100-7	0	54.0 (100)	0	36.0 (100)	7.0	3.2
0-0-10	51.5	0 (0)	34.0	0 (0)	10.0	4.5
50-0-10	27.8	27.7 (50)	34.0	0 (0)	10.0	4.5
90-0-10	5.5	46.0 (90)	34.0	0 (0)	10.0	4.5
100-0-10	0	51.5 (100)	34.0	0 (0)	10.0	4.5
0-50-10	51.5	0	17.0	17.0 (50)	10.0	4.5
0-90-10	51.5	0	5.4	30.6 (90)	10.0	4.5
0-100-10	51.5	0	0	34.0 (100)	10.0	4.5
50-50-10	27.8	27.7 (50)	17.0	17.0 (50)	10.0	4.5
90-90-10	5.5	46.0 (90)	5.4	30.6 (90)	10.0	4.5
100-100-10	0	51.5 (100)	0	34.0 (100)	10.0	4.5
100-100-20	0	44.0 (100)	0	27.0 (100)	20.0	9
100-100-30	0	36.5 (100)	0	20.0 (100)	30.0	13.5

^*^Superplasticiser additive/cement ratio = 0.008

^**^Effective water/cement ratio= 0.45. RAs were used under pre-saturation condition

### Characterisation techniques

The particle size distribution was studied by means of a mechanical shaker using Granutest model sieves (9.50 mm, 8 mm, 6.70 mm, 4.76 mm, 2.40 mm, 1.00 mm, 0.60 mm, 0.30 mm, 0.15 mm and 0.075 mm). The mineralogical characterisation of the CDW, the recycled aggregates (fine and coarse) and the natural aggregates (sand and gravel) was carried out using the XRD (X-ray diffraction) technique in a Shimadzu diffractometer model XRD 6000, with Cuα radiation and operating at 1.2 kW (40 kV e 30 mA). The diffractograms were registered in the interval of 5–60° 2θ, with a step size of 1°/min. The main elements in the CDW and in the natural and recycled aggregates were examined using the energy dispersive X-ray fluorescence (EDXRF) technique in a Bruker S2 Ranger LE spectrometer fitted with a 50 W X-ray tube (50 kV, 2 mA), Pd anode, XFlash® silicon drift detector with <135 eV resolution for Mn Kα and 100.000 cps, and equipped with a Peltier type cooling system (liquid nitrogen is not required) and primary filter tool changers with 9 positions possible. The trace elements were measured by inductively coupled plasma mass spectrometry (ICP-MS) by using an HP computer model HP4500®. The equipment was pre-calibrated with suitable standards.

The consistency (self-compacting) of fresh concrete was calculated using a V-funnel test as established in the UNE-EN 12350-9 (2011) standard. In order to determine the physical properties such as water absorption (WA), apparent porosity (AP) and bulk density (BD) of the hardened specimens, tests were performed in accordance with the UNE-EN 12390-3 (2020) standard for concrete, and the UNE-EN 1338 (2004) standard for concrete block paving.

The test specimens and paving units were immersed in water at a temperature of 20 ± 5 °C until they reached constant mass (immersed mass). Then, each specimen was dried using a cloth until the surface of the concrete is dull (wet mass). Finally, the materials were dried inside an oven at a temperature of 105 ± 5 °C until they reached a constant mass (dry mass). WA, BD and AP were determined according to the following equations:

1 $$ WA\ \left(\%\right)=\frac{\left({m}_w-{m}_d\right)}{m_d}\times 100 $$

2 $$ AP\ \left(\%\right)=\frac{\left({m}_w-{m}_d\right)}{\left({m}_w-{m}_i\right)}\times 100 $$

3 $$ BD\ \left( kg\ {m}^{-3}\right)=\frac{m_d}{\left({m}_w-{m}_i\right)} $$

where *m*_*w*_ is the wet mass, *m*_*d*_ is the dry mass and *m*_*i*_ is the immersed mass. Furthermore, in the case of regular and rectangular pavers, the best method of determining the BD is by using the *m*_*d*_/mass volume ratio (volume of solid, open a close porosity), which is calculated from the measured dimensions (ISO 5016 1997).

For measuring specific gravity (SG), the samples were finely ground (< 62 μm) in order to open the entire closed porosity. A weighted mass from this powder was used to determine its true volume and, therefore, its true density, by displacing distilled water inside a pycnometer (ISO 5018 1983).

4 $$ SG\ \left( kg\ {m}^{-3}\right)=\frac{\mathrm{Powder}\ \mathrm{Dry}\ \mathrm{Weight}}{\mathrm{True}\ \mathrm{Volume}} $$

The mechanical properties (compressive and tensile splitting strength) of the samples were compared with the properties of standards (without CDW), determined according to the requirements and test methods established in the UNE-EN 1338 (2004) standard, and performed with an EMIC apparatus, model DL-2000 at 7 and 28 days of curing. The mechanical test is carried out once the previous physical properties of the samples have been determined. The first mechanical test to be performed was the tensile splitting strength (*T*) test.

5 $$ T=0.637\ast k\ast \frac{P}{S} $$

where *T* (MPa), *P* is the measured load at failure (*N*), *S* is the area of failure plane (mm^2^) and *k* is a correction factor (*k*=0.87) (UNE-EN 1338 2004). Finally, the two parts of the samples retained from the tensile splitting strength test were tested in compression strength (σ) using the formula:

6 $$ \sigma =\frac{P}{A} $$

where *σ* (MPa), *P* is the measured load at failure (*N*) and *A* is the resisting area (mm^2^).

## Results and discussion

### Raw materials characterisation

**Fig. 2:**
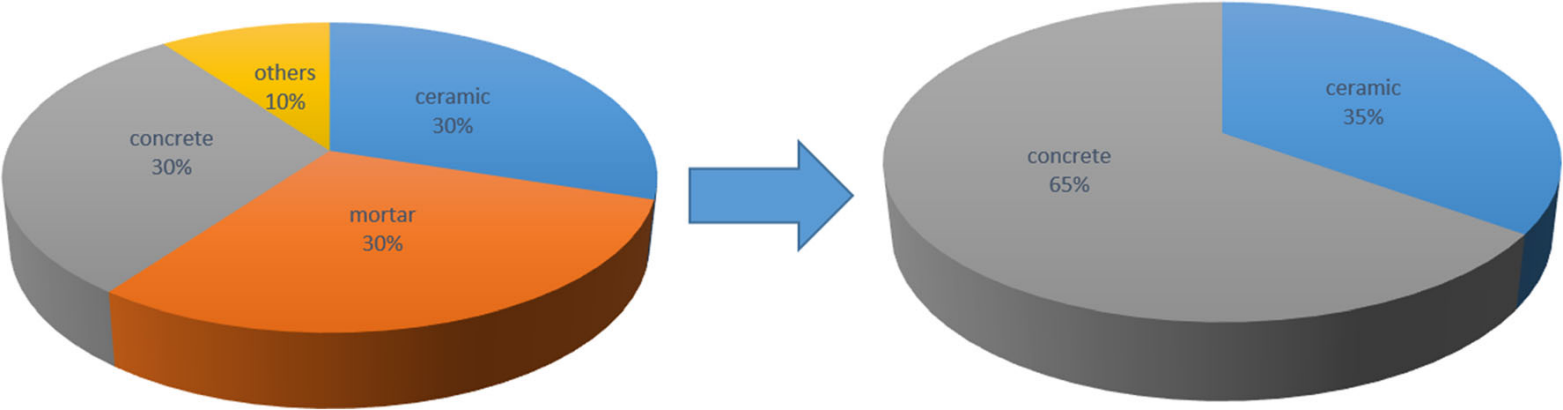
Material composition of CDW before (left) and after (right) the mechanical treatment

Attending to Fig. 2, CDW used in this study are mainly composed by ceramic (30 wt.%), concrete (30 wt.%), mortar (30 wt.%) and others (10 wt.%). The morphology of CDW was very irregular with a multitude of planes and angles. From the point of view of its resistance to fragmentation, the coarser particles are surrounded by parts that disaggregate when a force is applied to them, reducing the mechanical resistance. Therefore, a separation process was carried out intended to eliminate those fragile particles. As a result, the percentage of hard fractions in the sample increases: concrete aggregates (65 wt.%) and ceramic (35 wt.%).

**Fig. 3:**
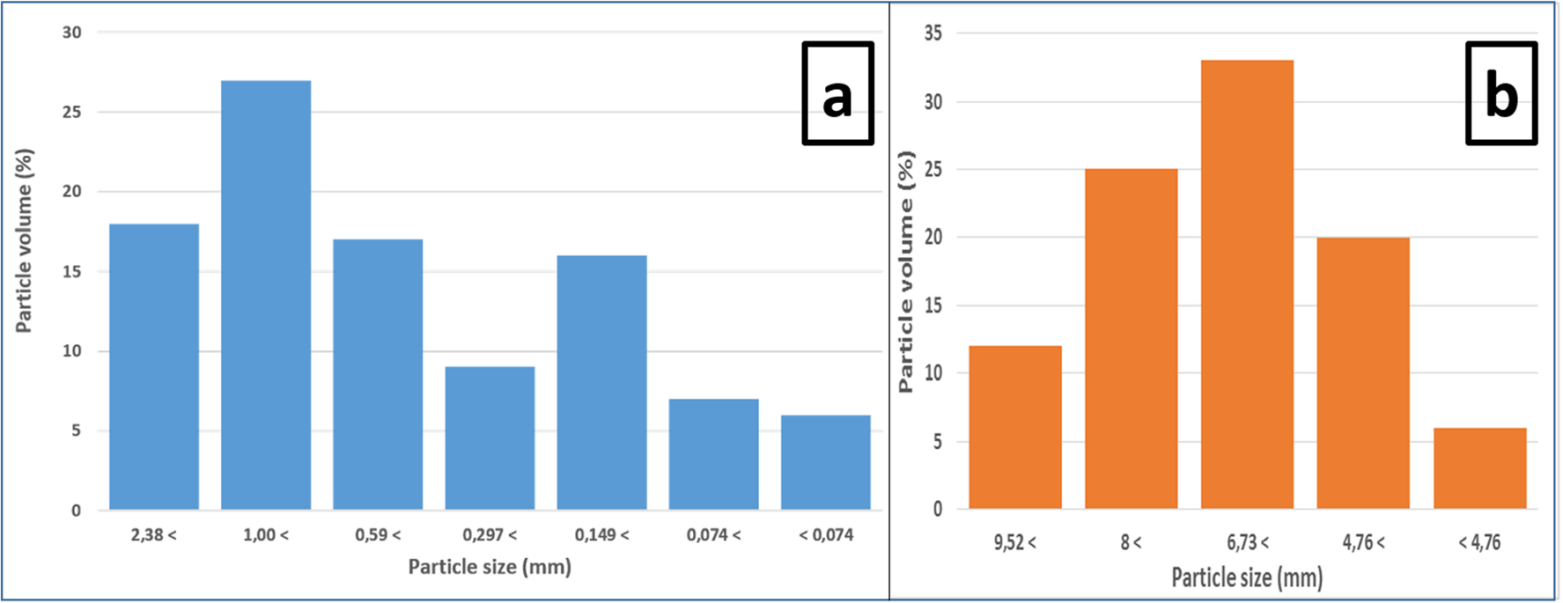
Particle size distribution of FRA (**a**) and CRA (**b**)

Figure 3 presents the results of the particle size analysis of RA. The particle size profile of the FRA indicated that the sample exhibited an asymmetric granulometric distribution with a broad interval of particle sizes; therefore, it can be assumed that it is a sandy material (from 4 to 0.075 mm). Figure 3a shows two main populations of particle size. The first population corresponds to particles with an average diameter of about 149 μm. The intermediate particle size fraction is the largest in this sample, with most particles having an average size of 1 mm. Mixing particles with different sizes improves particle packing, decreases porosity and water absorption, and increases concrete density (Shi et al. 2016; Tam et al. 2007). The particle size study of CRA is shown in Fig. 3b. It presented a symmetrical distribution with a wide range of particle sizes in the interval 4.75–9.52 mm, so CRA can be considered as gravel (from 4 to 20 mm). Figure 3b denotes as main populations between 6.3 and 8 mm in diameter.

The major elemental analysis by XRF indicated a vast array of elements in CDW composition (Table 2), mainly containing Si (75.5 wt.% as SiO_2_), Al (9.8 wt.% as Al_2_O_3_), Ca (6.1 wt.% as CaO), Fe (3.2 wt.% as Fe_2_O_3_), Mg (1.7 wt.% of MgO) and Ti (1.1 wt.% of TiO_2_). These results are similar to those found in the treated CDW fractions, whose main constituent is SiO_2_, 73.0 and 78.4 wt.% in CRA and FRA respectively. Regarding the NA, sand is mainly composed of SiO_2_ (90.6 wt.%) in the quartz form and Al_2_O_3_ (3.8 wt.%), according to the mineralogical study, while the gravel is mainly composed of SiO_2_ (63.2 wt.%), CaO (10.5 wt.%), Al_2_O_3_ (4.8 wt.%) and Fe_2_O_3_ (3.0 wt.%). On the other hand, Portland cement type I is composed of clinker and gypsum and mainly contains CaO, SiO_2_, Al_2_O_3_ and Fe_2_O_3_ (around 60, 21, 5 and 3 wt.%, respectively).

**Table 2:** Average concentrations expressed in oxides (*n* = 10) of major elements (% by weight) by XRF. Uncertainties given as standard deviation of the mean: *u* = (*S*_x_/*n*^1/2^), being *S*_x_ the standard deviation of the samples

	Na_2_O	MgO	Al_2_O_3_	SiO_2_	P_2_O_5_	SO_3_	K_2_O	CaO	TiO_2_	Mn_2_O_3_	Fe_2_O_3_	LOI
CDW	0.4 ± 0.1	1.7 ± 0.3	9.8 ± 0.5	75.5 ± 7.2	0.04 ± 0.01	0.2 ± 0.1	1.0 ± 0.2	6.1 ± 0.5	1.1 ± 0.2	0.05 ± 0.02	3.2 ± 0.4	5.1 ± 0.5
CRA	0.3 ± 0.1	1.4 ± 0.2	6.8 ± 0.6	73.1 ± 6.1	0.06 ± 0.02	0.2 ± 0.1	1.2 ± 0.2	6.4 ± 0.6	0.4 ± 0.1	0.06 ± 0.02	2.3 ± 0.2	5.7 ± 0.3
FRA	-	1.2 ± 0.2	5.4 ± 0.5	78.4 ± 4.9	0.06 ± 0.02	1.0 ± 0.2	0.8 ± 0.2	5.5 ± 0.4	0.2 ± 0.1	0.05 ± 0.01	1.9 ± 0.1	4.9 ± 0.4
Gravel	1.5± 0.3	1.4 ± 0.2	4.8 ± 0.5	63.2 ± 3.8	0.24 ± 0.08	0.6 ± 0.2	0.8 ± 0.1	10.5 ± 0.4	0.5 ± 0.1	0.05 ± 0.01	2.9 ± 0.2	1.9 ± 0.3
Sand	0.6 ± 0.2	1.3 ± 0.2	3.8 ± 0.4	90.6 ± 4.2	-	-	0.4 ± 0.1	1.5 ± 0.3	0.4 ± 0.1	0.20 ± 0.03	1.2 ± 0.1	0.7 ± 0.1
Cement	0.2 ± 0.1	2.2 ± 0.4	5.9 ± 0.5	21.5 ± 1.4	-	2.0 ± 0.4	0.7 ± 0.1	59.8 ± 1.1	-	-	2.8 ± 0.4	3.7 ± 0.3
Soil (*)	2.9	3.7	14.2	61.7	0.16	0.2	2.7	3.4	0.7	0.21	12.1	-

^*^Continental crust composition (Rudnick and Gao 2003)

Furthermore, the loss on ignition (LOI) in CDW and RA ranged from 4.9 to 5.7 wt.% (Table 2), and it was mainly associated with the release of volatiles; the liberation of water from hydrated lime and hydrated calcium silicates; the emission of carbon dioxide from carbonates; and the loss of water from phyllosilicates and other minor minerals present in CDW (Sharma and Goyal 2020; Zhang et al. 2017). In addition, the increased CaO is associated with the occurrence of CaCO_3_ in the RA, which also leads to increased LOI values, as its thermal decomposition produces CO_2_ emission.

In order to study the contaminants existing in the CDW, the trace elements (below 0.1 wt.%) were studied by ICP-MS (Table 3). The main trace elements identified were of the same order of magnitude as an unperturbed soil (Rudnick and Gao 2003). Similar results to CDW were obtained by FRA and CRA. Consequently, they do not present dangerous metals for both the materials and human health.

**Table 3:** Average concentrations (*n* = 10) of trace elements (mg kg^−1^). Uncertainties given as standard deviation of the mean: *u* = (*S*_x_/*n*^1/2^), being *S*_x_ the standard deviation of the samples

	Ba	Zr	V	Cr	Y	Rb	Zn	Cu	Sr	Pb	As
CDW	483 ± 30	385 ± 40	81 ± 14	58 ± 12	12 ± 4	32 ± 8	88 ± 5	80 ± 4	177 ± 11	28 ± 4	3.8 ± 0.3
CRA	405 ± 50	390 ± 24	101 ± 12	90 ± 21	11 ± 2	50 ± 6	45 ± 9	81 ± 6	230 ± 16	22 ± 3	3.9 ± 0.3
FRA	529 ± 40	385 ± 30	67 ± 9	89 ± 13	22 ± 4	45 ± 4	80 ± 7	89 ± 9	301 ± 18	26 ± 3	4.0 ± 0.9
Soil (*)	584	203	97	92	21	78	67	28	348	17	4.8

^*^Continental crust composition (Rudnick and Gao 2003)

**Fig. 4:**
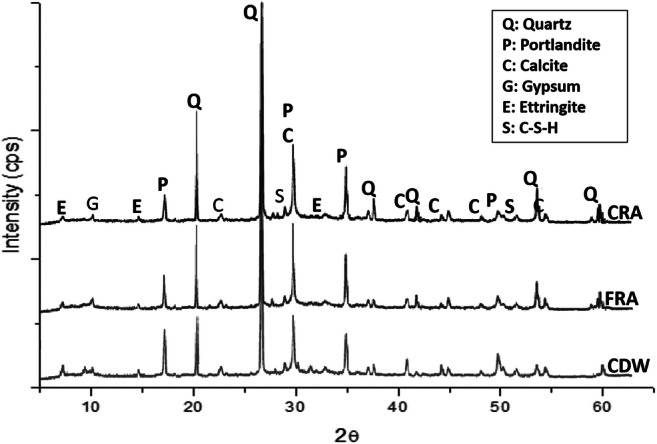
XRD pattern of CDW, FRA and CRA

According to the XRD analysis (Fig. 4), the CDW showed a complex mineralogical composition. This is associated with the large diversity of components contained in them, which include both amorphous and crystalline phases (coarse gravel or crushed rocks, sand, lime, cement, fired clay minerals, etc.) (Malhotra and Mehta 1996). CDW are mainly composed of quartz (SiO_2_), calcite (CaCO_3_) and portlandite (Ca(OH)_2_). In addition, the diffractograms indicate the presence of minor phases such as calcium silicate hydrate or C-S-H (3CaO·2SiO_2_·3H_2_O), gypsum (CaSO_4_·2H_2_O) and ettringite (Ca_6_Al_2_(SO_4_)_3_(OH)_12_·26H_2_O). These results agree with those reported in other studies (Contreras et al. 2016; Menezes et al. 2002; Saiz-Martínez et al. 2016). Furthermore, the XRD of the RA obtained by the CDW treatment revealed the same mineralogical composition than CDW. However, an increase in the intensity of peaks associated with quartz was observed. In the opposite way, the intensity of calcite and portlandite were reduced and even the low intensity peaks (C-S-H, gypsum and ettringite) almost disappeared.

**Fig. 5:**
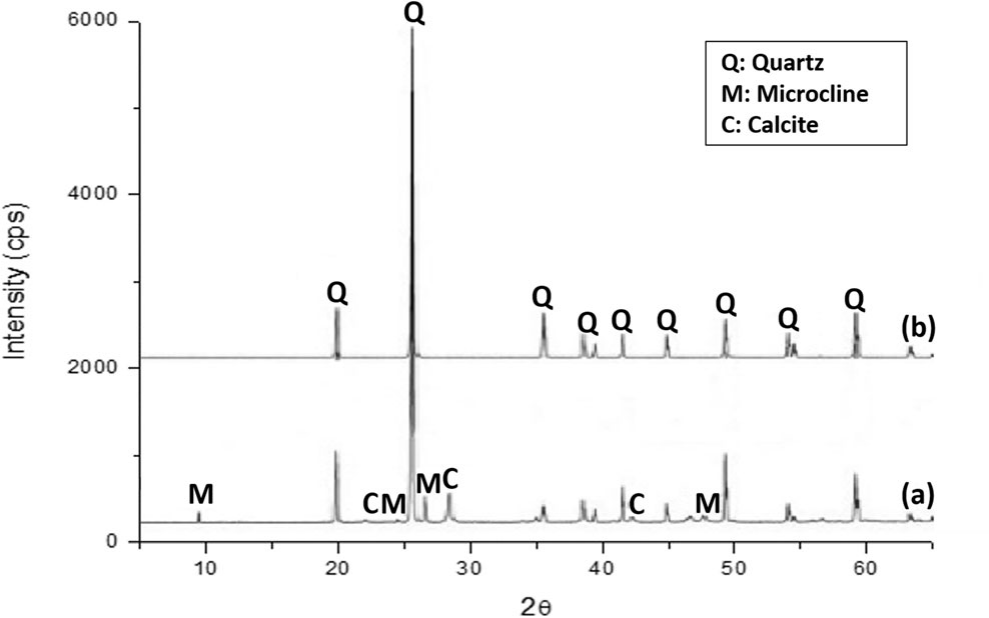
XRD of natural sand (**a**) and gravel (**b**)

On the other hand, the natural sand used is mainly composed of quartz (Fig. 5a), and some low intensity peaks in the XRD pattern denote the presence of calcite and to a lesser extent feldspar, such as microcline (KAlSi_3_O_8_). Gravel only includes quartz as crystalline phase (Fig. 5b).

Table 4 indicates that water absorption percentage (WA) of CDW was 7.6%. Furthermore, it also reveals that the WA of both FRA and CRA (4.8 and 5.0%, respectively) were quite lower than CDW although higher than NA (sand and gravel), which were 0.8 and 1.3% respectively. By comparing the analysis results with the WA limits from UNE-EN 12620 + A1 (2009), which specifies the required properties of natural, mechanically processed, recycled or mixtures of aggregates to be used in concrete, it can be said that both RA, in their current state, presented WA values within the limits recommended by EHE-08 (below 5.0%). The increased WA values of RA when compared to NA could be attributed to their higher porosity. The density of NA is in the order of magnitude of ∼ 2500 kg m^−3^, whereas the RA similar, ∼ 2300 kg m^−3^, by considering the experimental uncertainties, regardless of the type of CDW. The UNE-EN 12620 + A1 (2009) requires aggregates with SG greater than 2000 kg m^−3^. Consequently, these two fractions (FRA and CRA) met this requirement (see Table 4). In light of the above, the presence of attached mortar and ceramic materials in the RA caused a reduction in density and increase in WA in comparison with NA.

**Table 4:** Average (*n* = 10) physical characteristics (specific gravity-SG, bulk density-BD and water absorption-WA) of aggregates. Uncertainties given as standard deviation of the mean: *u* = (*S*_x_/*n*^1/2^), being *S*_x_ the standard deviation of the samples

	Sand	Gravel	CDW	FRA	CRA
SG (kg m^−3^)	2530 ± 190	2620 ± 140	2130 ± 180	2320 ± 120	2430 ± 140
BD (kg m^−3^)	1650 ± 50	1600 ± 60	1305 ± 30	1505 ± 50	1510 ± 50
WA (%)	0.8 ± 0.1	1.3 ± 0.1	7.6 ± 0.8	4.8 ± 0.3	5.0 ± 0.4

### Environmentally friendly concretes characterisation

Once the RA and the natural constituents were characterised, cylindrical concrete tests specimens (Fig. 1a) containing CRA and FRA (Table 1) were manufactured and tested after 28 days of curing (Table 5) according to the established requirements. The physical and technological properties were summarised in Table 5.

**Table 5:** Physical (specific gravity-SG, apparent porosity-AP and water absorption-WA) and technological (compressive strength-*σ* and tensile splitting strength-*T*) properties for each environmentally-friendly concrete composition (after 28 days of curing). Results show average values of 10 measurements of cylindrical test specimens. Uncertainties given as standard deviation of the mean: *u* = (*S*_x_/*n*^1/2^), being *S*_x_ the standard deviation of the samples

	Abrams (cm)	WA (%)	AP (%)	SG (g cm^-3^)	*σ* (MPa)	*T* (MPa)
0-0-7	11.3 ± 0.4	4.0 ± 0.4	8.5 ± 0.7	2.4 ± 0.2	20.5 ± 0.4	2.6 ± 0.2
10-0-7	10.1 ± 0.6	4.2 ± 0.4	8.9 ± 0.5	2.4 ± 0.2	20.9 ± 0.6	2.5 ± 0.3
25-0-7	10.8 ± 0.4	4.76 ± 0.7	9.5 ± 0.4	2.3 ± 0.2	20.6 ± 0.4	2.4 ± 0.3
50-0-7	11.9 ± 0.6	5.7 ± 0.8	9.6 ± 0.5	2.2 ± 0.2	19.3 ± 0.7	2.3 ± 0.2
75-0-7	11.4 ± 0.3	6.2 ± 0.6	10.8 ± 0.6	2.2 ± 0.2	18.5 ± 0.5	2.3 ± 0.3
90-0-7	12.8 ± 0.5	6.3 ± 0.8	10.7 ± 0.4	2.1 ± 0.3	18.3 ± 0.6	2.2 ± 0.2
100-0-7	10.7 ± 0.4	6.6 ± 0.6	11.0 ± 0.5	2.2 ± 0.2	18.1 ± 0.3	2.2 ± 0.2
0-10-7	12.1 ± 0.2	4.1 ± 0.4	8.9 ± 0.9	2.4 ± 0.2	20.4 ± 0.6	2.5 ± 0.3
0-25-7	9.6 ± 0.6	4.2 ± 0.4	10.5 ± 0.4	2.3 ± 0.1	20.2 ± 0.4	2.4 ± 0.3
0-50-7	11.4 ± 0.3	5.0 ± 0.7	9.6 ± 0.4	2.3 ± 0.2	18.6 ± 0.5	2.2 ± 0.1
0-75-7	10.8 ± 0.6	4.89 ± 0.4	9.5 ± 0.6	2.2 ± 0.2	18.3 ± 0.7	2.2 ± 0.2
0-90-7	11.2 ± 0.7	5.1 ± 0.8	11.0 ± 0.7	2.2 ± 0.2	17.6 ± 0.8	2.2 ± 0.3
0-100-7	10.5 ± 0.4	5.5 ± 0.6	10.2 ± 0.5	2.1 ± 0.2	17.5 ± 0.4	2.1 ± 0.3
10-10-7	11.9 ± 0.5	4.3 ± 0.4	9.2 ± 0.4	2.3 ± 0.2	20.2 ± 0.6	2.4 ± 0.2
25-25-7	12.1 ± 0.4	4.9 ± 0.4	8.5 ± 0.7	2.4 ± 0.2	20.0 ± 0.3	2.2 ± 0.2
50-50-7	9.8 ± 0.3	5.9 ± 0.6	9.1 ± 0.4	2.2 ± 0.2	19.1 ± 0.4	2.2 ± 0.3
75-75-7	10.2 ± 0.6	6.3 ± 0.4	9.5 ± 0.7	2.2 ± 0.3	18.2 ± 0.8	2.2 ± 0.2
90-90-7	11.9 ± 0.2	6.5 ± 0.5	10.9 ± 0.4	2.2 ± 0.2	17.1 ± 0.5	2.1 ± 0.3
100-100-7	10.7 ± 0.4	6.7 ± 0.4	11.0 ± 0.5	2.1 ± 0.2	17.2 ± 0.6	2.1 ± 0.3
0-0-10	11.2 ± 0.3	3.9 ± 0.6	9.4 ± 0.8	2.4 ± 0.1	22.5 ± 0.4	3.0 ± 0.2
50-0-10	11.5 ± 0.5	5.0 ± 0.6	8.9 ± 0.7	2.2 ± 0.2	21.2 ± 0.6	2.4 ± 0.4
90-0-10	11.0 ± 0.3	5.1 ± 0.6	9.1 ± 0.7	2.2 ± 0.2	20.1 ± 0.3	2.3 ± 0.4
100-0-10	12.3 ± 0.6	5.3 ± 0.7	11.0 ± 0.9	2.1 ± 0.3	20.3 ± 0.3	2.1 ± 0.3
0-50-10	11.2 ± 0.3	5.3 ± 0.5	10.2 ± 0.6	2.3 ± 0.2	20.5 ± 0.5	2.8 ± 0.3
0-90-10	10.2 ± 0.3	5.4 ± 0.5	11.1 ± 0.7	2.1 ± 0.2	20.4 ± 0.4	2.6 ± 0.3
0-100-10	11.2 ± 0.4	5.4 ± 0.8	11.0 ± 0.4	2.0 ± 0.3	20.2 ± 0.4	2.5 ± 0.5
50-50-10	12.1 ± 0.7	5.5 ± 0.7	10.0 ± 0.3	2.3 ± 0.2	20.6 ± 0.5	2.6 ± 0.4
90-90-10	10.2 ± 0.5	6.3 ± 0.8	11.3 ± 0.4	2.2 ± 0.2	19.3 ± 0.3	2.5 ± 0.4
100-100-10	10.9 ± 0.3	6.4 ± 0.6	11.2 ± 0.4	2.1 ± 0.1	19.6 ± 0.6	2.0 ± 0.7
100-100-20	12.2 ± 0.4	6.2 ± 0.6	10.6 ± 0.5	2.3 ± 0.3	21.5 ± 0.7	2.4 ± 0.3
100-100-30	10.1 ± 0.7	6.1 ± 0.7	10.1 ± 0.6	2.2 ± 0.1	26.3 ± 0.8	2.7 ± 0.8

**Fig. 6:**
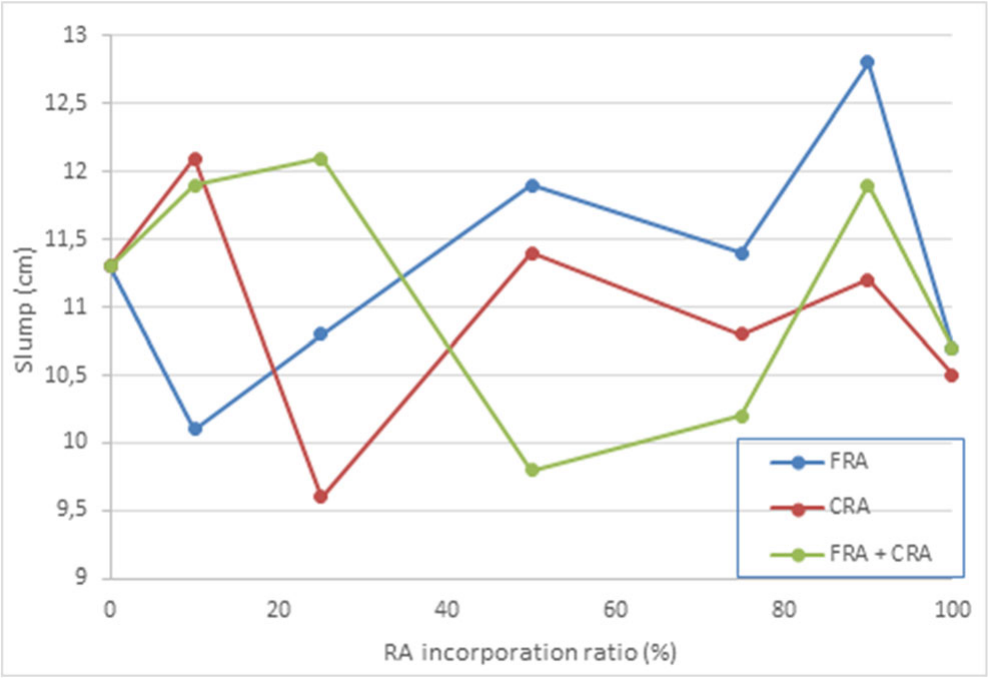
Test for workability of the fresh eco-friendly concrete

Before forming the cylindrical concrete specimens, the consistency was studied by the Abrams cone according to the UNE-EN 1250-9 2011. Table 5 shows all the values obtained from the slump test, these do not follow any trend either with the addition of cement or with the incorporation of RA. The water release by the RA may have been responsible for the slump test not following any trend and presenting high values. Moreover, Fig. 6 allows us to conclude that the viability of the various mixtures was kept within the consistency range (9.6 to 12.8 cm). These values were in accordance with other researches (Bermejo et al. 2010; Carro-López et al. 2018; Mefteh et al. 2013).

Moreover, the results obtained in the different tests carried out on cured concrete (Table 5) show that the replacement of NA by FRA and CRA affect the final properties of the concrete. In general, the addition of CDW increases water absorption (WA) and apparent porosity (AP), and antagonistic, it reduces both density (SG) and resistance (compressive strength (σ) and tensile splitting strength (T)). For this reason, these properties will be specifically analysed.

**Fig. 7:**
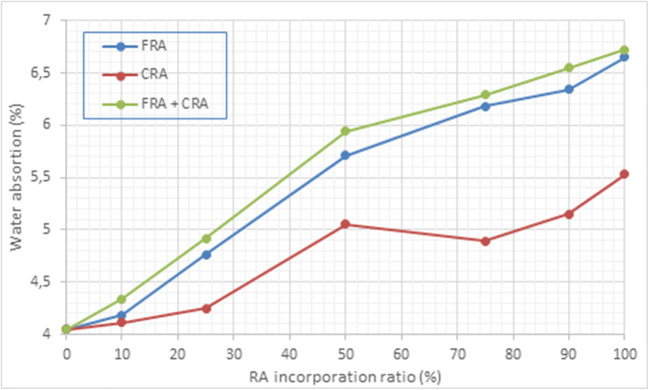
Graphical representation of water absorption vs. RA incorporation ratio

The WA of hardened concrete obtained according to UNE-EN 12390-7 (2020) increases with the incorporation of the RA, being higher when the grain size of the RA is smaller (Fig. 7). In addition, the finer particles occupied the pores and most of the external surface of the cylindrical specimens; therefore, WA was increased with the incorporation of the FRA fraction. On the other hand, WA values decreased considerably with the increase in the percentage of cement because the cementitious matrix increased, and therefore, the volume of pores present in the specimens was reduced. The WA values range from 4.0% in the control specimen (0-0-7) to values slightly higher than 6.5% (100-0-7 and 100-100-7). Although these values decreased with the increase of cement in the mixture, even with the highest concentration of RA, reaching values close to 6.0% (100-100-30).

The AP results shown in Table 5 indicate that the values ranged from 8.5 to 11.3%. The AP increased as the percentage of RA increased, especially with the incorporation of FRA. This physical property is crucial, since it is related to the water absorption of the cylindrical specimens (Bermejo et al. 2010; Gómez-Soberón 2002; Kumar and Bhattacharjee 2003; Moon and Moon 2002). Therefore, this property follows the same trend that water absorption (Fig. 7), because both properties are directly related.

Similar to the properties previously studied, the density (SG) increased lightly in the specimens with the highest cement content (Table 5), because their density is higher than that of RA and likewise because the cement occupied the open pores. On the other hand, density decreased with the incorporation of RA. This can be observed by comparing the result of the control specimen (0-0-7) of 2.4 g cm^−3^, with those with the highest concentration of FRA (100-0-7) of 2.2 g cm^−3^, and finally with those contain 100 wt.% CRA (0-100-7) of 2.1 g cm^−3^.

**Fig. 8:**
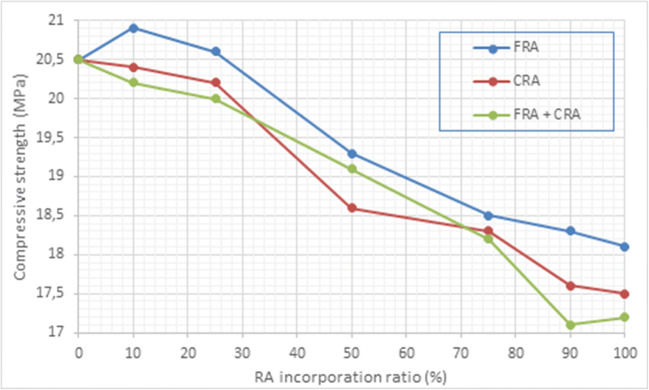
Graphical representation of compression strength vs. RA incorporation ratio

The most relevant technological properties for assessing the performance of a concrete structure are compressive strength (*σ*) and tensile splitting strength (*T*), as they are closely related to its potential to support stresses over time without failure. Therefore, both strengths allow a general assessment of the quality of the new concrete. Table 5 presents the evolution of compressive strength at 28 days (UNE-EN 12390-4 2020) as a function of the proportion of RA introduced into the concrete composition. It can be observed that regardless of the type and granulometry of the recycled aggregate, the resistance decreased in the samples with RA replacement by over 25 wt.% (Fig. 8).

According to the results of *σ*, samples containing up to 25 wt.% of RA showed similar values to those of the control specimen (ranging from 20.0 to 20.9 MPa) although the best results were achieved by the specimens incorporating up to 25 wt.% FRA (Falek et al. 2017; Idagu 2017; Tabsh and Abdelfatah 2009; Sharmal and Singla 2014; Silva et al. 2014). In contrast, lower strength values were obtained for replacements greater than 25 wt.%, achieving a 15% reduction in the compressive strength of the materials prepared by complete replacement of NA by RA. The heterogeneous composition of the RA and the increase in total water/cement ratio due to the saturation of the RA necessary for their incorporation into the concrete were responsible for these reductions in compressive strength.

On the other hand, the specimens with a higher proportion of cement achieved the best resistance results. This statement can be validated by observing how the compressive strength values increase with increasing cement in the mixture: 17.2 MPa (100-100-7), 19.6 MPa (100-100-10), 21.5 MPa (100-100-20) and 26.3 MPa (100-100-30), Table 5.

**Fig. 9:**
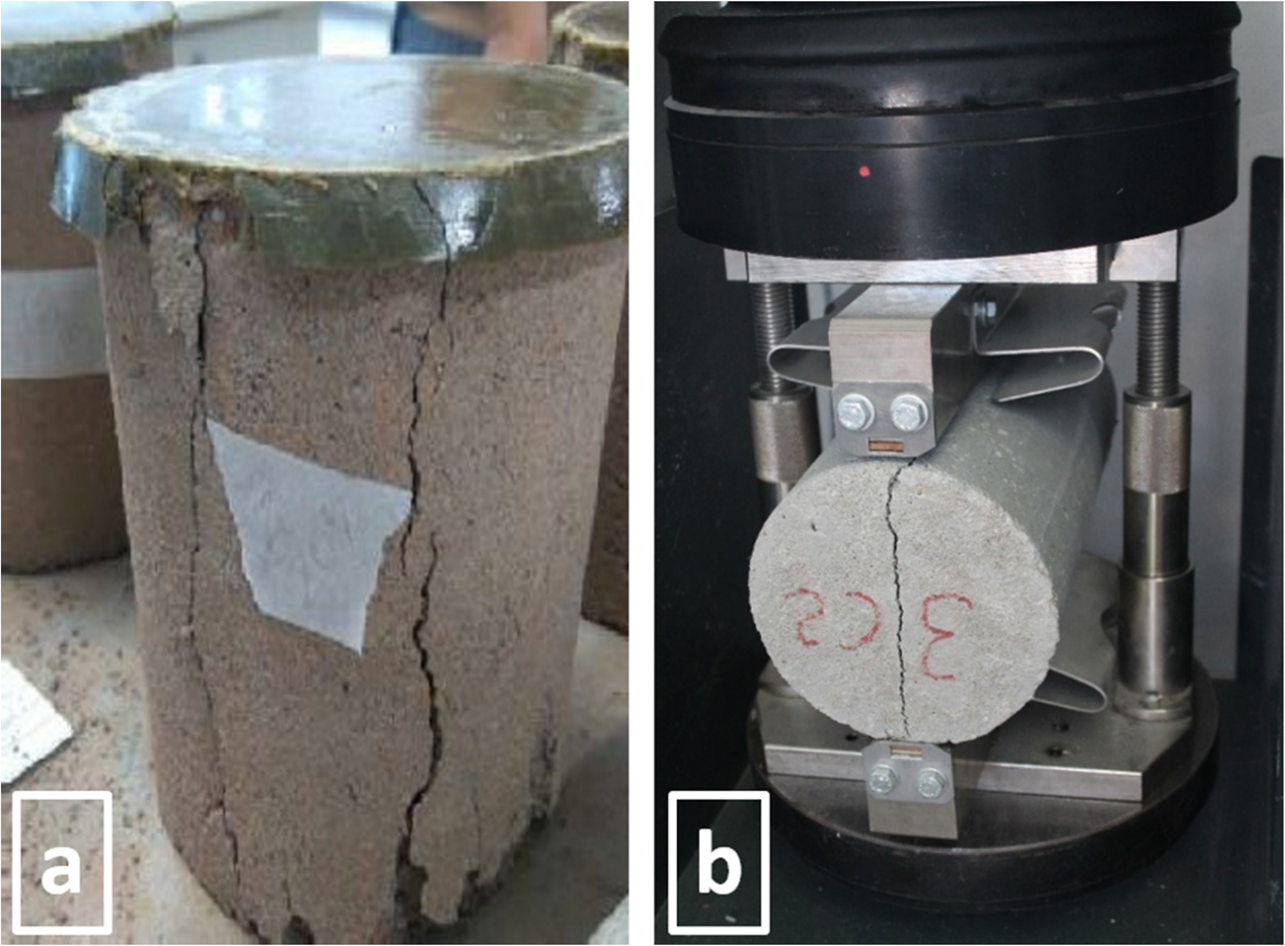
Exhibiting prismatic fractures of the specimens after compression (**a**) and tensile splitting (**b**) strengths test

Observation of the concrete specimens after the compressive strength test leads to the inference that the load distribution during the test (Fig. 9a) was homogeneous, as the specimens exhibited a characteristic prismatic fracture.

**Fig. 10:**
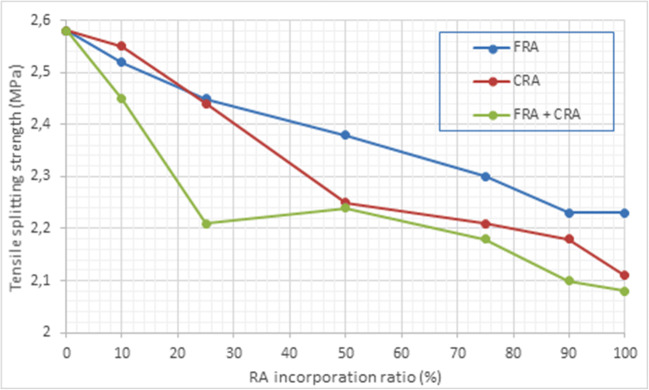
Graphical representation of tensile splitting strength vs. RA incorporation ratio

Moreover, the characteristic tensile splitting strength at 28 days for concrete made with RA (CRA and/or FRA) is shown in Table 5. The concrete specimens show values in the range 2.5–2.1 MPa, while the reference material reached an average value of 2.6 MPa. Therefore, a decrease of approximately 0–20% in the tensile splitting strength of concrete elements with the incorporation of RA was observed (Fig. 10). However, concrete specimens processed by replacing 25 wt.% of NA with FRA show comparable strength values (less than 5% variance) to those of concrete produced with NA. The presence of bonded mortar, ceramic materials, etc., in the RA may have been responsible for the observed loss in tensile splitting strength. Furthermore, as in the previous case, it should be noted that the increase in the cement concentration in the mixture increased the *T* of the specimens, as it is expected.

The cylindrical specimens after the tensile splitting strength test showed almost perfect longitudinal fracture, which is characteristic of samples subjected to a uniform longitudinal load distribution during the test (Fig. 9b).

### Paving blocks characterisation

After studying the influence of the incorporation of the RA in the production of environmentally friendly concrete, the possibility of replacing NA with RA in the manufacture of constructive elements by using precast concrete elements such as paving block was evaluated (Fig. 1b), whose physical and technological properties (after 28 days of curing) are shown in Table 6.

**Table 6:** Physical (specific gravity-SG, apparent porosity-AP and water absorption-WA) and technological (tensile splitting strength-*T*) properties of the paver blocks manufactured with RA (after 28 days of curing). Results show average values of 10 measurements. Standard uncertainty calculated as the standard deviation of the mean

	WA (%)	AP (%)	SG (g cm^−3^)	*T* (MPa)
0-0-7	5.0 ± 0.8	8.7 ± 0.7	2.4 ± 0.2	3.9 ± 0.2
25-0-7	5.3 ± 0.5	9.1 ± 0.4	2.3 ± 0.2	3.9 ± 0.3
50-0-7	5.7 ± 0.9	9.2 ± 0.5	2.2 ± 0.2	3.7 ± 0.2
75-0-7	5.8 ± 0.4	9.8 ± 0.6	2.2 ± 0.2	3.4 ± 0.4
100-0-7	6.0 ± 0.7	10.0 ± 0.5	2.2 ± 0.2	3.1 ± 0.4
0-25-7	5.2 ± 0.4	9.5 ± 0.4	2.3 ± 0.1	3.9 ± 0.3
0-50-7	5.3 ± 0.7	9.9 ± 0.4	2.3 ± 0.2	3.6 ± 0.2
0-75-7	5.9 ± 0.4	10.5 ± 0.6	2.2 ± 0.2	3.1 ± 0.4
0-100-7	6.5 ± 0.6	11.2 ± 0.5	2.1 ± 0.2	2.9 ± 0.4
25-25-7	5.9 ± 0.4	9.5 ± 0.7	2.4 ± 0.2	3.6 ± 0.3
50-50-7	6.9 ± 0.6	10.1 ± 0.4	2.2 ± 0.1	3.5 ± 0.3
75-75-7	7.3 ± 0.4	10.5 ± 0.7	2.1 ± 0.3	3.0 ± 0.5
100-100-7	8.2 ± 0.3	11.0 ± 0.5	2.2 ± 0.2	2.9 ± 0.6

UNE-EN 1338 (2004) establishes two types of paving blocks, depending on the value of WA: WA < 6%, called class 2 and mark B, where the paving is frost resistant (this is the most demanding requirement) and the second if WA > 6%, called class 1 mark A. In this sense, paving blocks containing up to 75 wt.% of RA can be considered as class 2, mark B.

Table 6 summarises the results of tensile splitting strength determined for concrete paver blocks prepared with RA. Concrete pavers with up to 25 wt.% of RA (coarse and fine) showed an average tensile splitting strength of 3.9 MPa, the same value as that obtained for the reference material (0-0-7) (De Brito et al. 2005; Mas et al. 2012; Özalp et al. 2016; Poon and Chan 2007), exceeding the characteristic tensile splitting strength of 3.6 MPa established in the UNE-EN 1338 standard (2004), and the obtained results are not less than 2.9 MPa for any individual sample. Moreover, paving units containing 50 wt.% FRA or CRA were shown not to have a significant effect on the strength. On the other hand, paver blocks prepared with FRA above 75 wt.% replacement did not exceed the tensile splitting strength threshold of 3.6 MPa. However, this research concludes that concrete specimens formulated with FRA as a substitute for NA (up to 50 wt.%) or by a mixture of FRA and CRA (50 wt.% and 25 wt.%, respectively) comply the mechanical specifications for paver blocks. These results allow us to conclude the viability of replacing NA with RA in the manufacture of concrete paving blocks.

### Environmental implications

Throughout its service life, concrete has an environmental impact resulting from different factors such as the production of the raw materials, its manufacture, its use and maintenance throughout its service life and, finally, its demolition. In this assessment, we will focus on the impact associated with the production of raw materials, specifically aggregates, assuming that the other variables are not significantly influenced by the use of natural or recycled aggregates. According to Pimiento and Restrepo (2018), 0.008 tons of CO_2_ are emitted in the production of one ton of gravel or sand produced by open-cast mining, while this emission is reduced to 0.001 tons of CO_2_ emitted in the production of one ton of aggregates from CDW.

According to the dosage used in this study, in the manufacture of precast concrete elements (37.2 wt.% gravel, 55.8 wt.% sand, 7.0 wt.% cement) the CO_2_ emission associated with the extraction of the aggregates necessary to manufacture 1 ton of precast concrete can be estimated as 7.4 kg (Varela Alberte 2012). Considering the substitution of up to 50% of natural aggregate by recycled aggregate, in the manufacture of 1 ton of precast concrete elements with the incorporation of CDW, 4.18 kg of CO_2_ will be emitted, which means a reduction of 43.7% in the amount of CO_2_ released into the atmosphere (Varela Alberte 2012).

But in addition to the advantages derived from lower CO_2_ emissions, other environmental benefits associated with the replacement of natural aggregates with recycled aggregates must be taken into account, such as reduction of the volume of extraction of limited raw materials, thus preserving natural resources; reduction of mining waste generated in the extraction of reduction of landfill requirements for mining waste resulting from the extraction of mining waste; and reduction of landfill requirements for mining waste. Therefore, the precast concrete in this study can be considered as environmentally friendly. However, it is clear that, in order to ensure that the manufacture of concrete incorporating recycled aggregates is assumed by the industry, it is necessary to ensure competitive manufacturing costs. In this sense, the production cost of recycled aggregates is 15% lower than that of gravel extraction and 27% lower than that of sand extraction (Varela Alberte 2012). Therefore, its implementation at industrial level is also viable from an economic point of view.

## Conclusions

In this study, the use of recycled aggregates in the manufacture of concrete paving blocks was analysed. Thus, the variability in the composition, the lower density and the higher water absorption of CDW are aspects to be borne in mind. In this sense, the addition of FRA and CRA increases the water absorption (WA) and the apparent porosity (AP) of concrete, reducing both its density and resistance (compressive strength (*σ*) and tensile splitting strength (*T*)). Additions of up to 50 wt.% of fine (50-0-7) or coarse (0-50-7) recycled aggregates or the substitution of 25 wt.% of each (sample 25-25-7) result in materials with similar properties to the reference materials. Moreover, the results are in accordance with the values established by the EHE-08 for concrete manufacture.

Finally, the technological properties of paving blocks manufactured with up to 50 wt.% of RA replacement have a mechanical behaviour similar to the reference material (0-0-7). The water absorption below 6.0% (class 2 and mark B) and the characteristic tensile splitting strength above 3.6 MPa the minimum values established in the UNE-EN 1338 standard, which means that they can be used in pedestrian areas or areas with little traffic.

## Data Availability

Not applicable
